# Imposition Practiced by Dentists

**Published:** 1849-07

**Authors:** 


*Imposition Practiced by Dentists.*—Among the many proofs of malpractice which have come to our knowledge, we have seldom seen or heard of any more reprehensible than one which was mentioned to us, not long since, by a professional friend. He stated that he had recently been consulted by an individual who, a short time before, had had several of his teeth filled, but which had ever since been more or less troublesome, and occasionally sore to the touch and painful. Upon examination, he at once discovered that the caries had only been very partially removed, and the fillings introduced in a loose and imperfect manner. On making these facts known to his patient, he was requested to remove the old fillings and insert new ones. In the removal of the fillings which he found in his teeth, he discovered that they consisted principally of tin foil, the orifice only being filled with gold, and both so imperfectly consolidated that a common plugging instrument could, with ease, be readily passed through them.

To introduce gold into a tooth in this manner, is bad enough, as no good whatever can result from an operation thus performed, but to fill two-thirds of the cavity with tin, and the other third with gold, leading the patient to suppose that the last named metal was the only one employed, is a species of malpractice that cannot be too strongly censured. Fillings of bees-wax, would contribute quite as effectually to the preservation of teeth, and possess the advantage of being *rather* more easily introduced, while they would not be so likely to occasion uneasiness by exciting a galvanic action, which the contact of two different metals in the mouth is almost certain to do.—*Balt. Ed.*

